# A Heart too Stiff to Beat: A Case of Familial Transthyretin Amyloidosis Cardiomyopathy

**DOI:** 10.7759/cureus.1106

**Published:** 2017-03-20

**Authors:** Yan Zhou, Sameen Khalid, Aamer Abbass, Laura Hughes, Marcos Hazday

**Affiliations:** 1 GME, Florida Hospital-Orlando; 2 Internal Medicine Residency, Florida Hospital-Orlando; 3 Cardiology, Florida Hospital-Orlando

**Keywords:** transthyretin, amyloidosis, cardiomyopathy, liver transplant

## Abstract

Heart failure is a common clinical syndrome caused by a variety of cardiac diseases. We report a rare case of familial transthyretin amyloidosis cardiomyopathy to heighten the awareness of this rare but lethal cause of heart failure, as therapeutic interventions such as liver or heart transplant could be curative in selected patients.

## Introduction

Amyloidosis refers to the extracellular deposition of insoluble fibrils that are composed of low molecular weight subunits of a variety of serum proteins. The three most common types of amyloidosis that can affect the heart are light-chain (AL) amyloidosis, familial or senile transthyretin amyloidosis (ATTR) and secondary (AA) amyloidosis [1]. Transthyretin (TTR) is a tetrameric serum protein that is synthesized primarily by the liver. Accumulation of TTR causes cell toxicity and tissue death. ATTR occurs in two types: non-mutated (senile/wild type) or mutated transthyretin with autosomal dominant inheritance (familial type) [2]. Familial ATTR is caused by any one of more than 100 mutations in the TTR gene, rendering it unstable and resulting in a tendency to misfold and produce amyloid which preferentially infiltrates the myocardium [3]. One of the most common mutations is caused by the Val122Ile mutation, which presents in three to four percent of the African-American and Afro-Caribbean populations. It is associated with a late-onset amyloid cardiomyopathy (CMP) characterized by progressive severe heart failure [4-6]. We report a case of familial ATTR cardiomyopathy to heighten the awareness of this rare but lethal cause of heart failure. Informed consent statement was obtained for this study.

## Case presentation

**Figure 1:**
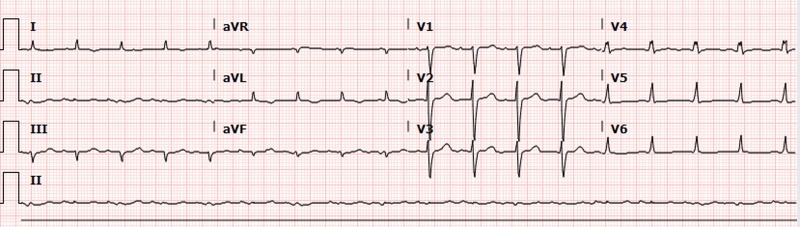
Electrocardiogram Low QRS voltage without ischemic changes

A 62-year-old Afro-Caribbean male was diagnosed with congestive heart failure with left ventricular ejection fraction (LVEF) 35% two years ago at his home country and presented with progressing shortness of breath, paroxysmal nocturnal dyspnea, orthopnea. Other comorbidities included well-controlled type two diabetes mellitus and hypothyroidism. His home medications included furosemide, metformin, and levothyroxine. He has two family members who passed away from unknown heart disease after their 50s. Vital signs were significant for chronic borderline hypotension. Examination showed elevated jugular venous pressure, decreased breath sounds at the left base, and S3 heart sound. The electrocardiogram (EKG) showed low QRS voltage, but no ischemic changes [Figure 1].

**Figure 2:**
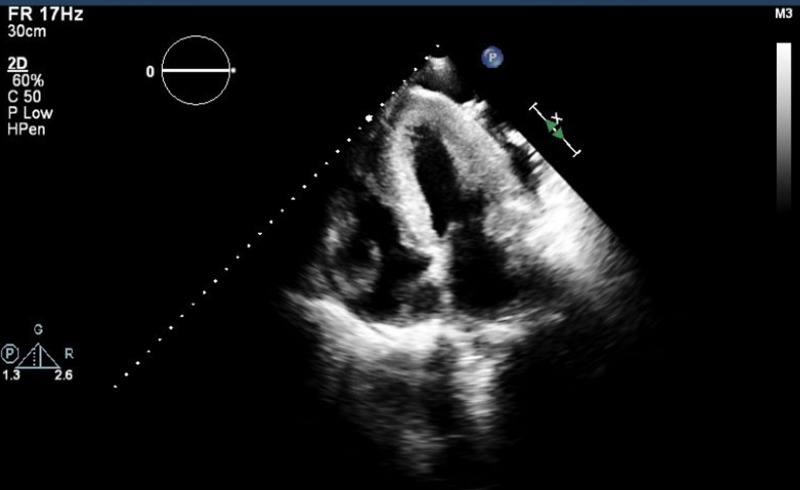
Transthoracic echocardiography four chamber view Severe concentric biventricular hypertrophy

Troponins were equivocal. The N-terminal pro-B-type natriuretic peptide (NT-proBNP) was elevated. Chest X-ray showed a moderate left pleural effusion with basilar atelectasis and mild pulmonary edema. Transthoracic echocardiography (TTE) revealed global hypokinesis with LVEF 16% and severe concentric biventricular hypertrophy [Figure 2].

**Figure 3:**
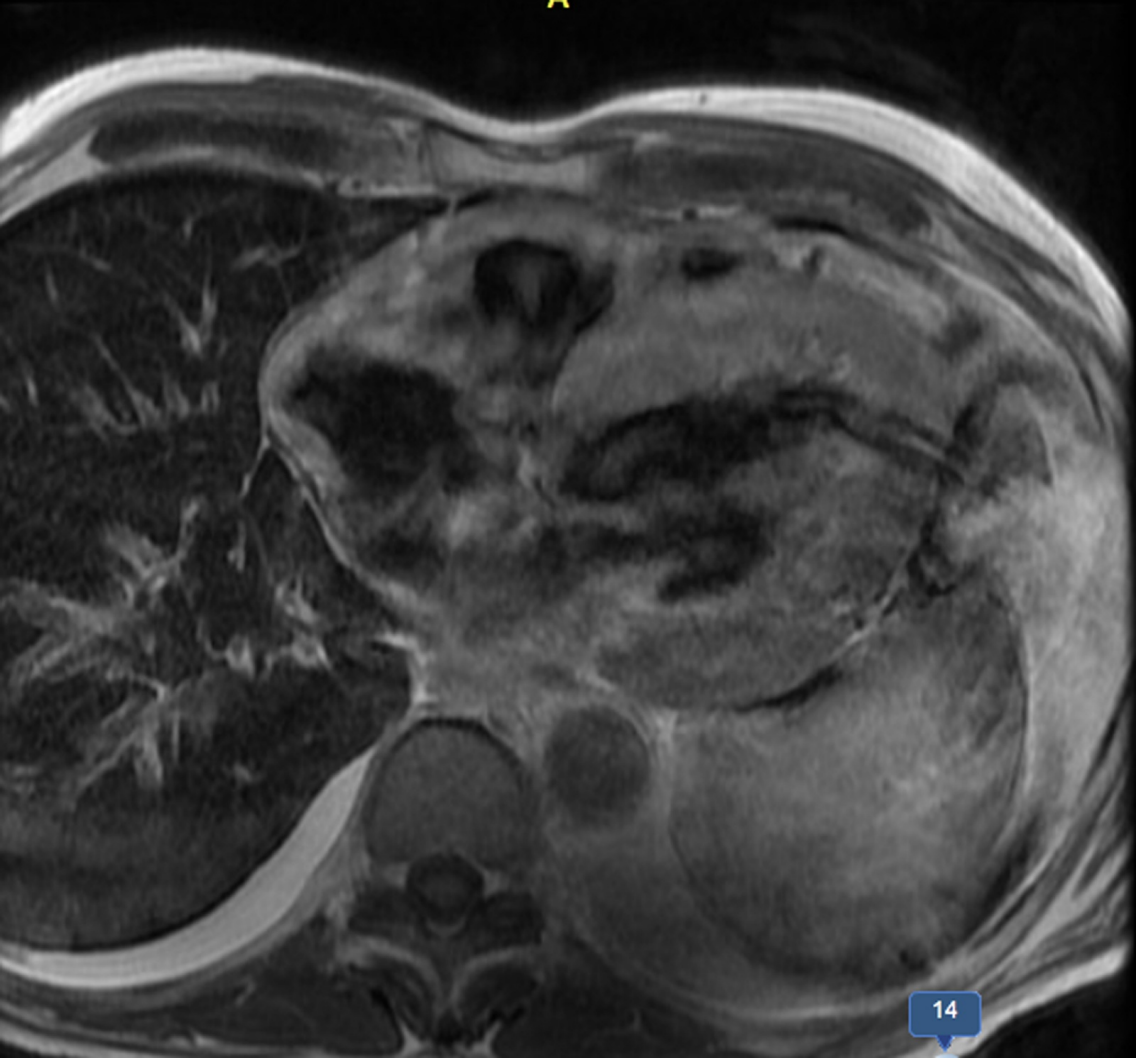
Cardiac MRI with late gadolinium enhancement Diffusely enlarged concentrically thickened ventricles with increased myocardial signal suggestive of amyloid deposition by appearance

The patient was started on IV diuretics, dobutamine drip and had left-sided therapeutic thoracentesis with symptomatic improvement. Cardiac magnetic resonance imaging (MRI) with late gadolinium enhancement revealed diffusely enlarged concentrically thickened ventricles with increased myocardial signal suggestive of amyloid deposition by appearance [Figure 3].

**Figure 4:**
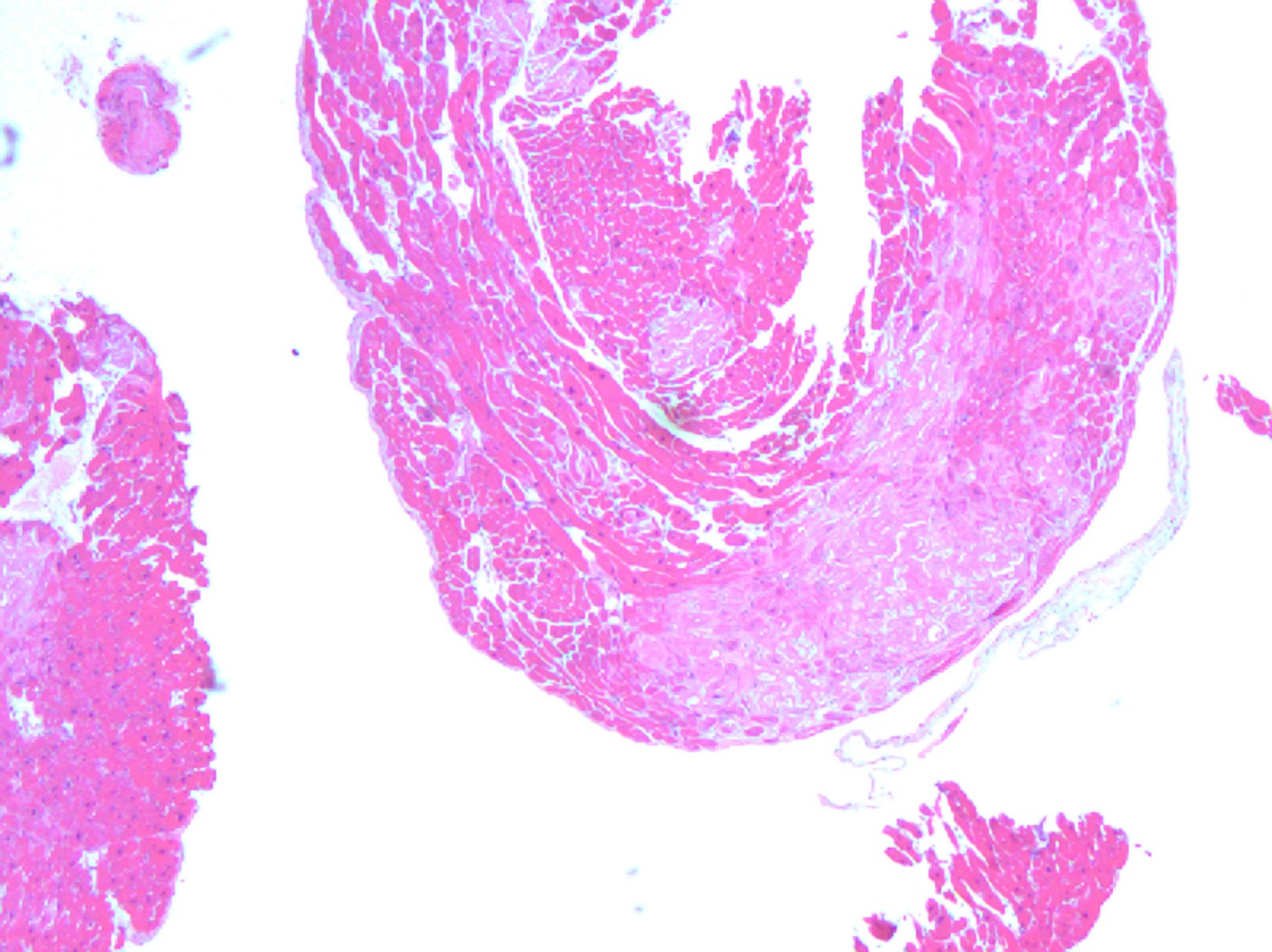
Haemotoxylin and eosin (H&E) staining

**Figure 5:**
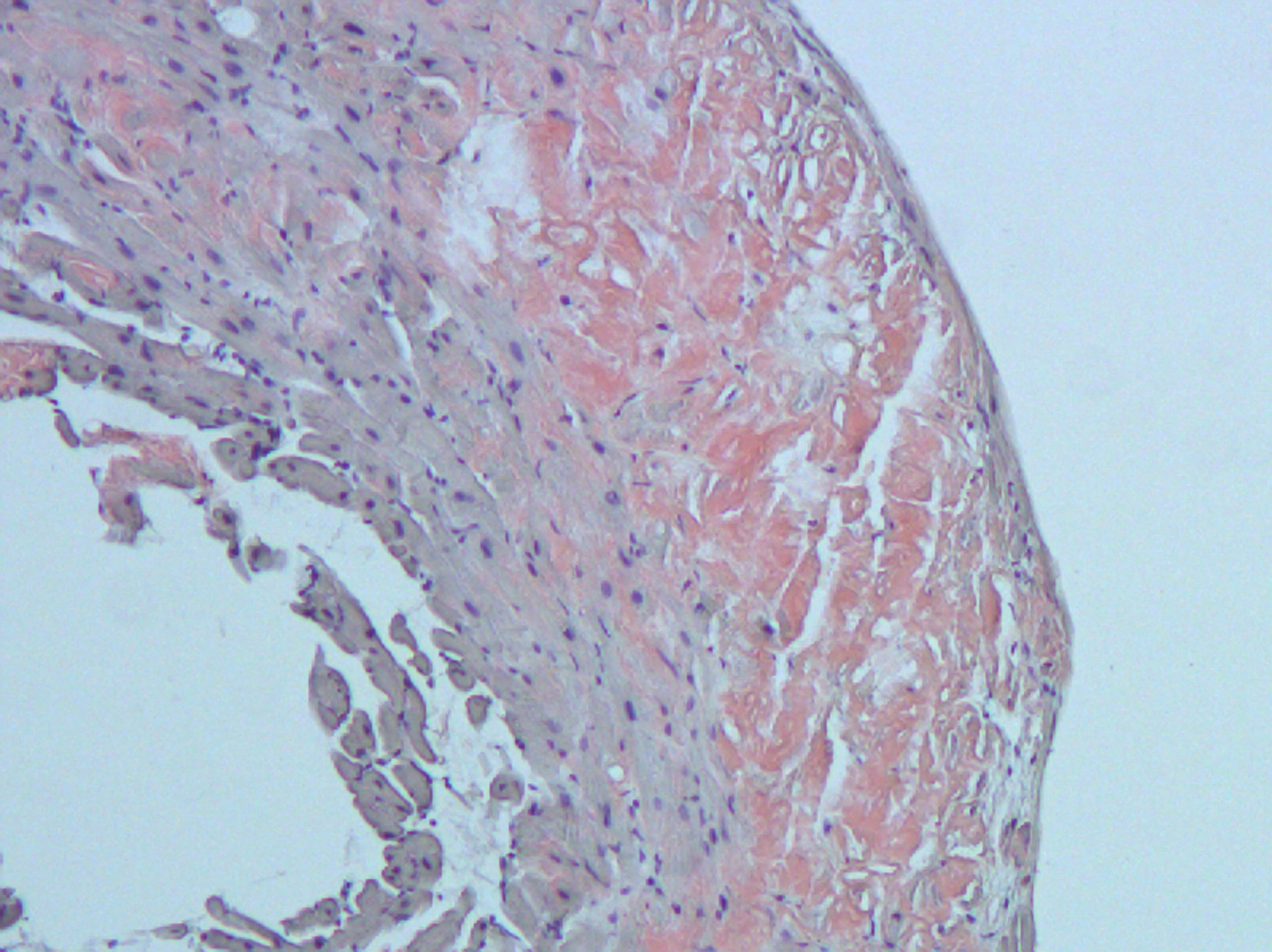
Congo red staining The myocardium contains large patches of hyaline material that stains with Congo red, consistent with amyloid

Left and right heart catheterization showed single-vessel coronary artery disases (CAD) isolated to the first obtuse marginal artery. Serum and urine immunofixations were negative. Right ventricular endomyocardial biopsy was obtained with amyloid subtype staining indicating ATTR [Figures 4-5].

Further genetic sequencing analysis showed pathogenic Val122Ile mutation and confirmed familial ATTR cardiomyopathy. The patient developed several runs of asymptomatic non-sustained ventricular tachycardia during the hospital stay. Due to high risk for deadly arrhythmias, the patient underwent single-chamber automatic implantable cardioverter-defibrillator (AICD) placement. He was started on warfarin for long-term anticoagulation due to nearly immobile atriums and ventricles. He was evaluated for heart and liver transplants. The liver transplant was postponed due to his heart condition, and ideally, should be pursued after the heart transplant in order to preserve the transplant heart from amyloid deposition. Due to severe hypertrophy with significantly decreased chamber size, left ventricular assisted device was not feasible. Eventually, he was deemed not to be a candidate for the heart transplant due to increased pulmonary vascular resistance. He is currently on palliative care.

## Discussion

In patients without hypertension or valvular disease, biventricular hypertrophy on TTE should raise suspicion for infiltrative cardiomyopathy particularly when associated with low voltage on EKG, as hypertensive left ventricular hypertrophy or hypertrophic cardiomyopathy will usually have high/normal QRS voltage. The cardiac MRI with late gadolinium enhancement is very helpful in supporting the diagnosis of amyloid cardiomyopathy with a sensitivity of 80% and specificity of 94%, positive and negative predictive values of 92% and 85%, respectively [7]. Endomyocardial biopsy is the biopsy of choice for diagnosing ATTR, due to high rates of negative biopsies elsewhere [2]. After histologic confirmation of amyloidosis, if special immunostaining to confirm amyloid subtype is not available, serum and urine immunofixation, immunoglobulin free light chain assay, and bone marrow biopsy are needed to exclude amyloid light-chain (AL) amyloidosis. Further TTR mutation sequencing analysis will differentiate familial from senile type ATTR. In terms of treatment, diuretics use is complicated in ATTR cardiomyopathy as stiff heart is often preload dependent. Beta blockers, angiotensin-converting-enzyme (ACE) inhibitors/ angiotensin receptor blockers (ARBs) should be used with caution because they are often poorly tolerated due to hypotension [2]. Early prophylactic AICD placement is rational, given the high incidence of sudden death from arrhythmia and electrical mechanical dissociation [8-9]. Heart and/or liver transplantation are the most important therapeutic interventions. Liver transplantation can be curative in selective patients as it removes the nidus of mutant TTR and prevents recurrent amyloid deposition. More research is needed to evaluate potential pharmacological agents on stabilizing amyloidosis [2]. 

## Conclusions

We presented a rare case of familial ATTR cardiomyopathy. Unfortunately, by the time of definitive diagnosis, the patient was no longer a candidate for heart or liver transplant, which are potentially curative interventions. Assessment of African American or Afro-Caribbean patients with unexplained heart failure should include a differential diagnosis of transthyretin amyloidosis, as early diagnosis is important to initiate appropriate therapeutic interventions.
